# Optimisation of Lactobacillus fermentation conditions and its application in the fermentation of salt-free sauerkraut

**DOI:** 10.3389/fmicb.2024.1482163

**Published:** 2024-10-21

**Authors:** Wenlun Wang, Wenbing Li, Yan Huang, Ying Yang, Hui Liu, Chaohang Yu, Qing Yuan, Lianmin He, Qianmin Hu, Ye Li, Taoyan Meng, Huanhuan Chen, Jiabi Liao, Ou Chen, Shirui Yu, Feng Zhang

**Affiliations:** ^1^Department of Food Science and Engineering, Moutai Institute, Zunyi, China; ^2^Department of Biology and Chemistry, College of Liberal Arts and Sciences, National University of Defense Technology, Changsha, China

**Keywords:** salt-free sauerkraut, lactic acid bacteria, probiotics, Plackett-Burman experiment, central composite design

## Abstract

To identify what are the dominant lactic acid bacteria (LAB) involved in the fermentation of salt-free sauerkraut, and optimize its industrial culture conditions, we isolated and identified a strain of LAB, which is referred to as Lactobacillus sp. DF_001, with the preservation number CCTCC NO: M20232593, from five different regions in Guizhou Province. Industrial culture conditions were optimized using Plackett-Burman and Central Composite design experiments, and the potential role of this LAB in salt-free sauerkraut fermentation was validated. Bioproduction was optimal with a culture time of 66 h, starch/water ratio of 1.7% and inoculum of 0.02%, which gave approximately three-fold higher yield than the basal culture medium DeMan-Rogosa-Sharpe medium (MRS). The LAB was used in small-scale industrial experiments. The Dafang LAB significantly enhanced the sensory score of the salt-free sauerkraut products by about 32% compared to the control group. The total acid content increased by about 32% and the sugar and nitrite contents were reduced by 67.27 and 69.58%, respectively. The total number of bacterial colonies decreased by 37.5%. All other indicators complied with the national standard, providing overall the basis to improve salt-free sauerkraut fermentation.

## 1 Introduction

Sauerkraut, commonly referred to as pickle, is a type of fermented vegetable that has been a staple in Chinese cuisine for over 3,000 years (Peng et al., 2018). In Guizhou province, its production can be of two types. The first type is salted and fermented, as is made for example in Dushan County (Mou, 1989), and similar fermentation methods are used for northeastern and Sichuan sauerkrauts (Ouyang et al., 2019). However, these sauerkrauts contain nitrite which may lead to health problems when consumed in large quantities and over a long period (Wang Y. et al., 2019). As a consequence, there is a global growing demand for alternative industrial fermentation methods.

The second type of fermented sauerkraut in Guizhou Province is salt-free, where production methods follow those used by farmers to make homemade water sauerkraut. This type relies on natural fermentation under anaerobic conditions, following microbial enrichment with lactic acid bacteria (LAB) and other beneficial microorganisms, which reduces nitrite content and enhances acidity and taste. Indeed, it has been shown that LAB naturally degrade sauerkraut nitrites, affecting texture and sensory properties (Tian et al., 2023). And the use of salt-free sauerkraut, known for its high cellulose content, appetising flavour and greasy texture, together with optimization of the use of LAB, should prevent the problems associated to salt-cured sauerkraut. Therefore, selecting dominant LAB is crucial for enhancing the industrial fermentation of salt-cured sauerkraut.

LAB have a range of applications in food, agriculture, chemical industry or medicine (Wang J. et al., 2019), and include Gram-positive genera such as Lactobacillus or Bifidobacterium, among others (Wang J. et al., 2019; Liu et al., 2016; Cani et al., 2022). LAB are found in fermented foods such as sauerkraut (Van Zyl et al., 2020), yogurt, acid cabbage and other pickled products (Ağagündüz et al., 2021). They are considered to be probiotic, preventing intestinal infection (Li et al., 2021) and having a general beneficial effect on health (Garbacz, 2022). Because of these probiotic properties, LAB are used in the fermentation of vegetables such as mustard tuber, kohlrabi, north-eastern sauerkraut and peppers (Ye et al., 2018). Flavor, quality and nutritional value in sauerkraut is affected by the type and quantity of LAB used (Yifei X. et al., 2021). And in salt-free fermented sauerkraut (Lin et al., 2021), mutual interaction of a diverse set of bacteria, with a major role of LAB, results in sauerkraut with a characteristic texture. Commercial production of salt-free sauerkraut requires industrial LAB cultivation, but this is affected by high turbidity and excessive suspended matter (Zhang, 2021; He J. et al., 2021; Jing et al., 2016), negatively impacting flavour and taste, later stages of fermentation or strain management (Zuo et al., 2022; Kim et al., 2022; Yang et al., 2016). Moreover, a review of the literature indicates a scarcity of studies focused on optimizing industrial cultivation conditions for LAB. Thus, optimizing and characterizing the industrial cultivation conditions of LAB for sauerkraut fermentation in Guizhou Province is crucial.

In the present paper, we explored water sauerkraut in five regions of Guizhou Province to identify a strain of dominant LAB (Lactobacillus sp. DF_001, with the preservation number CCTCC NO: M20232593). Industrial cultivation of this strain was optimized and its effect on the fermentation of salt-free sauerkraut was tested. We hypothesize our results can be used to overcome common problems in the industrial cultivation of LAB, and at the same time improving quality and taste of salt-free sauerkraut, enhancing acidity and reducing sugar and nitrite content. Implementation of these results may result in the successful commercialization of industrially made salt-free sauerkraut.

## 2 Materials and methods

### 2.1 Materials, isolation and identification of bacteria

The T-AOC Assay Kit and DPPH Free Radical Scavenging Assay Kit were purchased from Beijing Solarbio Science & Technology Co., Ltd., China. A total of 100 colorimetric test assay kits and nitric oxide metabolite assay kits were purchased from Shanghai Merck Science & Technology Co., Ltd., China. A total cholesterol (TC) colorimetric assay kit was purchased from Shanghai Elabscience Science & Technology Co., Ltd., China. MRS broth and LB broth were purchased from Beijing Solarbio Science & Technology Co., Ltd., China. All other chemicals were of analytical grade and were commercially available.

#### 2.1.1 DeMan-Rogosa-Sharpe medium (MRS)

The following composition of the MRS was used in this study: 10.0 g of peptone, 10.0 g of beef extract, 5.0 g of yeast extract, 2.0 g of diammonium hydrogen citrate, 20.0 g of glucose, 5.0 g of sodium acetate, 20 g of dipotassium hydrogen phosphate, 0.25 g of magnesium sulphate heptahydrate, 0.25 g of manganese sulphate, and 1 mL of Tween 80. Deionised water was added to reach a volume of 1,000 mL, and the mixture was sterilised at 121°C for 20 min before use. If solid medium was needed, 1.5–2.0% agarose was added, and the mixture was sterilised for later use.

Plate count agar (PCA) medium was prepared by the addition of 5 g of tryptone, 2.5 g of yeast extract, 1.0 g of glucose, and 15.0 g of agar to 1,000 mL of deionised water. The mixture was sterilised at 121°C for 20 min before use.

Lauryl sulphate tryptone (LST) broth was prepared by combining tryptone (20 g), sodium chloride (5 g), lactose (5 g), dipotassium hydrogen phosphate (2.75 g), and potassium dihydrogen phosphate (2.75 g). The final pH was adjusted to 6.8 ± 0.2 with lactic acid, after which the total volume was brought to 1,000 mL with deionized water. The broth was sterilised at 121°C for 20 min and used for the detection of coliform bacteria.

#### 2.1.2 Sample collection of salt-free sauerkraut juice

Samples were obtained from salt-free sauerkraut soup made by farmers from five counties in Guizhou Province: Qixingguan (longitude: 105.305, latitude: 27.298), Honghuagang (106.894, 27.645), Luban (106.402, 27.792), Nayong (105.383, 26.778), and Dafang (105.613, 27.142). The sauerkraut was thoroughly mixed in a fermentation container and a sterilised micropipette was used to draw a 40 mL sauerkraut juice sample which was placed in a sterilised 50 mL sampling tube. The sample was taken to the laboratory and refrigerated waiting for LAB purification.

#### 2.1.3 Isolation and purification of LAB

The samples were diluted (10^−1^ to 10^−8^) with 0.9% NaCl (normal saline). After shaking at 37°C overnight, 100 μL from the 10^−8^ well were spread on an MRS plate. The plates were cultured upside down at 37°C overnight. Single colonies were observed and colonies were selected. Single colonies were streaked on plates. Streak culture was performed 2–3 times to ensure strain purity. After isolation and purification, the strains were subjected to Gram staining and microscopic morphological examination, followed by inoculation into MRS broth tubes for subculture and used to streak again in MRS media.

#### 2.1.4 Isolation and identification of LAB

Identification of the strains was performed according to previous methods (Kim et al., 2022; Yang et al., 2016; Liu et al., 2021) with slight modifications. Phenotype and Gram staining were recorded, focusing on transparency, color, surface smoothness, unevenness, and wire drawing of the LAB. To identify the isolates, the TSINGKE Plant DNA Extraction Kit (Universal Type) was used following the manufacturer’s instructions. The extracted genomic DNA was stored at −20°C for subsequent PCR amplification tests. Purified polymerase chain reaction amplicons from the isolates were sequenced using the universal primers 27F (5`-AGTTTGATCMTGGCTCAG-3`) and 1492R (5`-GGTTACCTTGTTACGACTT-3`). For identification, 16S rRNA sequences were searched using BLAST (http://www.ncbi.nlm.nih.gov/BLAST, accessed October 1, 2023).

#### 2.1.5 Determination of the growth curve

The 5-bead local LAB strain was first activated for two generations. For each activation, 1.0% inoculum was added to MRS broth medium and cultured in a constant temperature vertical shaker at 37°C for up to 12 h. The blank control was not inoculated. For the MRS broth culture medium, liquid samples were taken every 3 h, measuring absorbance at 600 nm, and ensuring that absorbance was between 0.5 and 3.0 (typically diluting 3–5 times). This experiment was repeated three times to obtain growth curves of strains from different locations, plotting absorbance (y) as a function of culture time (x).

### 2.2 Determination of physical and chemical indicators

*Determination of nitrite content*: Nitrite content was determined with reference to GB 5009.33–2016 “National Food Safety Standard—Determination of Nitrite and Nitrate in Food”.^1^

*Determination of total acid content (calculated as lactic acid)*: Lactic acid content was determined with reference to GB 12456–2008 “National Food Safety Standard—Determination of Total Acid in Food”.^2^

*Determination of reducing sugar content:* Sugar content was determined with reference to GB/T 15038–2006 “National Food Safety Standard—Determination of Total Sugar in Food”.^3^

*Coliform group detection:* Coliform group was determined with reference to the GB/T 4789.3–2016 “Food Safety National Standard Food Microbial Inspection Coliform Group Count” (MPN) counting method for coliform group determination.^4^

*Detection of the total number of colonies:* The total number of colonies was determined according to GB 4789.2–2016 “National Food Safety Standard Food Microbiological Inspection Determination of Total Bacterial Colony”.^5^

*Absorbance measurements:* An ultraviolet (UV) spectrophotometer was used to measure the 600 nm absorbance and average OD_600_ was calculated. The number of LAB at 1OD was previously shown to be 1 × 10^8^ bacteria/mL (Bai et al., 2021).

*Determination of the number of LAB:* The total number of LAB was determined according to the methods provided in GB 4789.35–2023.^6^

*Measurement of Cell Surface Hydrophobicity:* Bacterial adhesion of LAB to hydrocarbons was measured as described (Kim et al., 2022; Yang et al., 2016; Liu et al., 2021) with slight modifications. Briefly, LAB isolates cultured overnight were centrifuged at 8000 g for 5 min. The pellet was washed twice with sterile phosphate-buffered saline (PBS; pH 7.2) and resuspended in sterile PBS to an optical density of 0.5 (A0) at 600 nm. The suspension was mixed vigorously with an equal amount of xylene (Sigma–Aldrich) and incubated at room temperature for 1 h. The separated aqueous phase was carefully removed, and its absorbance was measured (A1). Surface hydrophobicity (H%) was calculated using the formula H% = (1 − A1/A0) × 100%.

*Hydroxyl radical scavenging assay:* Determination of total antioxidant capacity (T-AOC) was performed using a commercial kit (Beijing Legen Biotechnology Co., Ltd.) according to the manufacturer’s instructions.

*DPPH radical scavenging assay*: This assay was performed according to the instructions of the DPPH free radical scavenging ability detection kit (Beijing Suolaibao Technology Co., Ltd.).

*Iron ion reducing capacity:* An iron ion reduction capacity kit (Beijing Suolaibao Technology Co., Ltd.) was used to determine the iron ion reducing ability of the SEOs.

*Determination of cholesterol content:* Total cholesterol (TC) content was measured using a cholesterol assay kit (Applygen Technologies, Beijing, China) according to the manufacturer’s instructions.

*Sensory Evaluation:* The sensory evaluation method was performed as previously described with minor modifications (Du et al., 2022; Zhao et al., 2022; Akomea-Frempong et al., 2021). The samples were evaluated for colour, texture, smell and crispness, by 11 trained evaluators and a minimum of 100 consumers. Each parameter was assigned a number from 1 to 10 (10 = like extremely and 1 = dislike extremely). Sensory evaluation details were determined according to T/GZSX023-2017 “Standards of Guizhou Food Industry Association”^7^ and DBS22/025–2014 “Local Standards for Food Safety (pickled cabbage)”.^8^

### 2.3 Optimum design of culture conditions

#### 2.3.1 Single-factor experimental design

To optimize the growth of LAB, single factor tests were performed varying starch leach solutions (wheat, potatoes, rice, sweet potatoes and corn), culture times (12 h, 24 h, 48 h and 72 h), starch/water ratios (0.5, 1, 1.5 and 2%) and inoculum (0.05, 0.1, 0.15 and 0.2%).

#### 2.3.2 Central composite design

The response pattern (Reddy et al., 2008) and the optimal combination of culture time, starch/water ratio and inoculum for maximising LAB yield were evaluated using a central composite design with three variables (Table 1). The Plackett-Burman design showed significant curvature and confirmed the importance of all three parameters. The variables that had the greatest potential for maximising LAB activity were selected as centre points for the central composite design. The experimental data were analysed using a predictive quadratic polynomial equation to establish a correlation between the response variable and the independent variables (Xie et al., 2014):


$$\begin{array}{l} Y=\alpha_{0}+\alpha_{1}X_{1}+\alpha_{2}X_{2}+\alpha_{3}X_{3}+\alpha_{11}X_{1}^{2}+\alpha_{22}X_{2}^{2}+\\ \alpha_{33}X_{3}^{2}+\alpha_{12}X_{1}X_{2}+\alpha_{13}X_{1}X_{3}+\alpha_{23}X_{2}X_{3} \end{array}$$

where Y is the predicted response; α_0_ is the intercept; α_1_, α_2_, and α_3_ are linear coefficients; α_11_, α_22_, and α_33_ are quadratic coefficients, and α_12_, α_13_ and α_23_ are interactive coefficients. The experimental design was developed using Design Expert 8.0.7.1 (Statease, Inc., Minneapolis, MS, United States).

**Table 1:** Central composite design experimental design.

Variable	Code	Level				
		−1.68	−1	0	1	1.68
Culture time (h)	A	7.64	24	48	72	88.36
Starch/water ratio (%)	B	−0.182	0.5	1.5	2.5	3.182
Inoculum (%)	C	−0.0018	0.005	0.015	0.025	0.0318

### 2.4 Small-scale fermentation experiment

**Figure 1:**
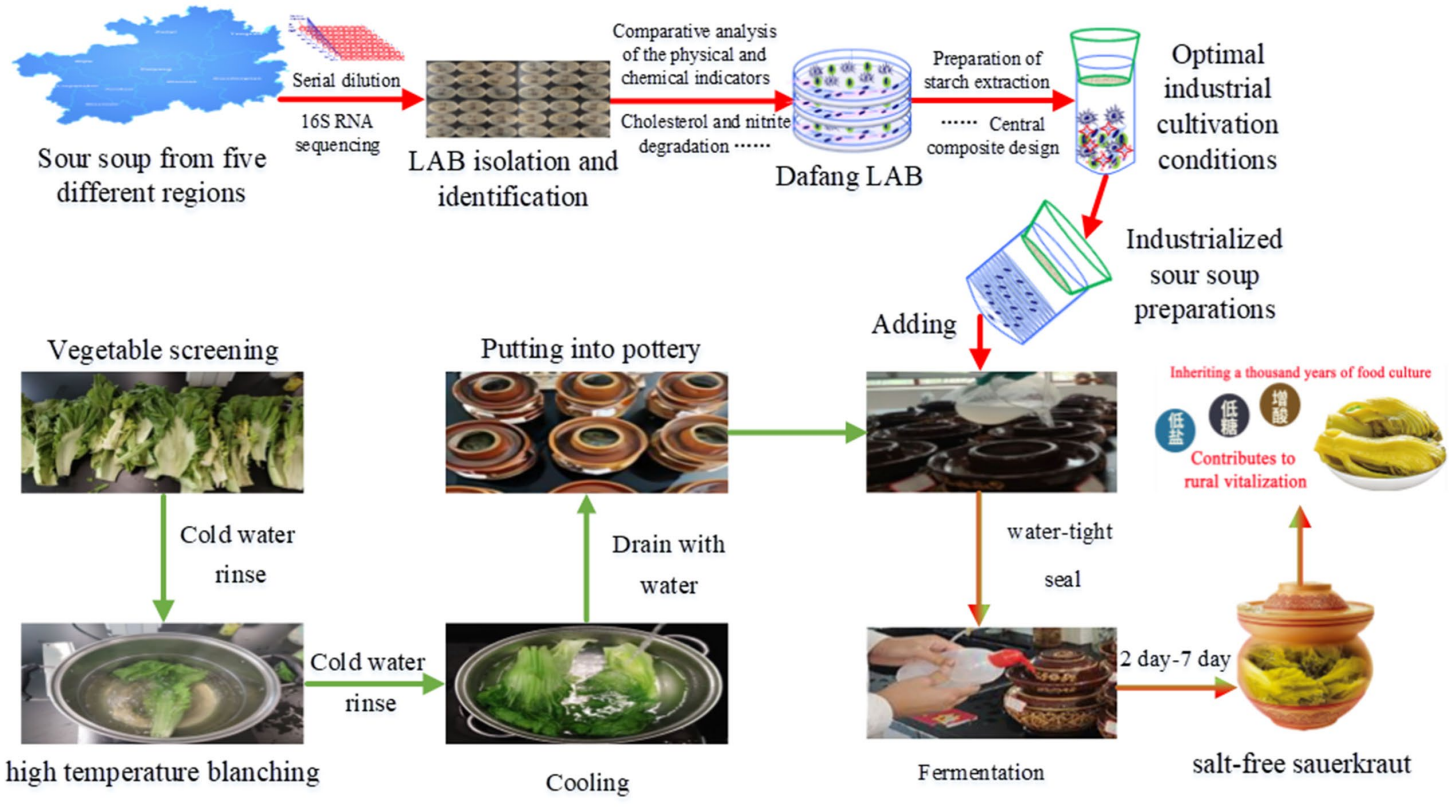
Flow chart of laboratory Shamita fermentation process.

Salt-free sauerkraut was produced in a small-scale fermentation experiment. Green vegetables were used as raw materials which were blanched for 30 to 60 s in a starch/water ratio of 1.71%, inoculated with 0.017% at 30–35°C and fermented for 7 days (Figure 1). Compliance with relevant standards was assessed using simulated pasteurisation, by heating in a water bath at 63°C for 15 min, followed by analysis of various parameters (Section 2.2 and related kit instructions).

### 2.5 Data processing and statistical analysis

All data were processed and visualised using Design Expert 13, GraphPad Prism 9, R language and Excel software. Experimental results are expressed as means ± standard error of the mean (mean ± SD). Standard error analysis was performed on the single-factor experimental samples. Significance analysis was performed using an internal function in Design Expert 13, and a significance level of *p* < 0.05 indicated significant differences.

## 3 Results

### 3.1 Separation and identification of LAB

The strains isolated from the five locations were all Gram-positive, appeared as long or short rods (Supplementary Figure S1) and were consistent with the morphological traits of LAB, as stated in the Bergey’s Manual of Determinative Bacteriology. Samples were then tested for homology using 16S rDNA. Comparison of the 16S rDNA sequences with BLAST using the NCBI database showed that all five strains belonged to *Lactobacillus fermentum* (referred to as Lactobacillus) within the Lactobacillus family, with a 100% homology (Table 2).

**Table 2:** Results of a BLAST search for the 16S rDNA sequences of all five isolates.

Sample	Accession	Kingdom	Phylum	Class	Order	Family	Genus	Reference species	Homology (%)
Qixing guan	MZ577210.1	Bacterial kingdom	Firmicutes	Bacillus	Lactobacillus	Lactobacillus	*Limosilacto bacillus*	*Limosilactobacillus fermentum*	100
Honghua gang	MZ577210.1	Bacterial kingdom	Firmicutes	Bacillus	Lactobacillus	Lactobacillus	*Limosilacto bacillus*	*Limosilactobacillus fermentum*	100
Luban	MZ577210.1	Bacterial kingdom	Firmicutes	Bacillus	Lactobacillus	Lactobacillus	*Limosilacto bacillus*	*Limosilactobacillus fermentum*	100
Nayong	MZ577210.1	Bacterial kingdom	Firmicutes	Bacillus	Lactobacillus	Lactobacillus	*Limosilacto bacillus*	*Limosilactobacillus fermentum*	100
Dafang	MZ577210.1	Bacterial kingdom	Firmicutes	Bacillus	Lactobacillus	Lactobacillus	*Limosilacto bacillus*	*Limosilactobacillus fermentum*	100

**Figure 2:**
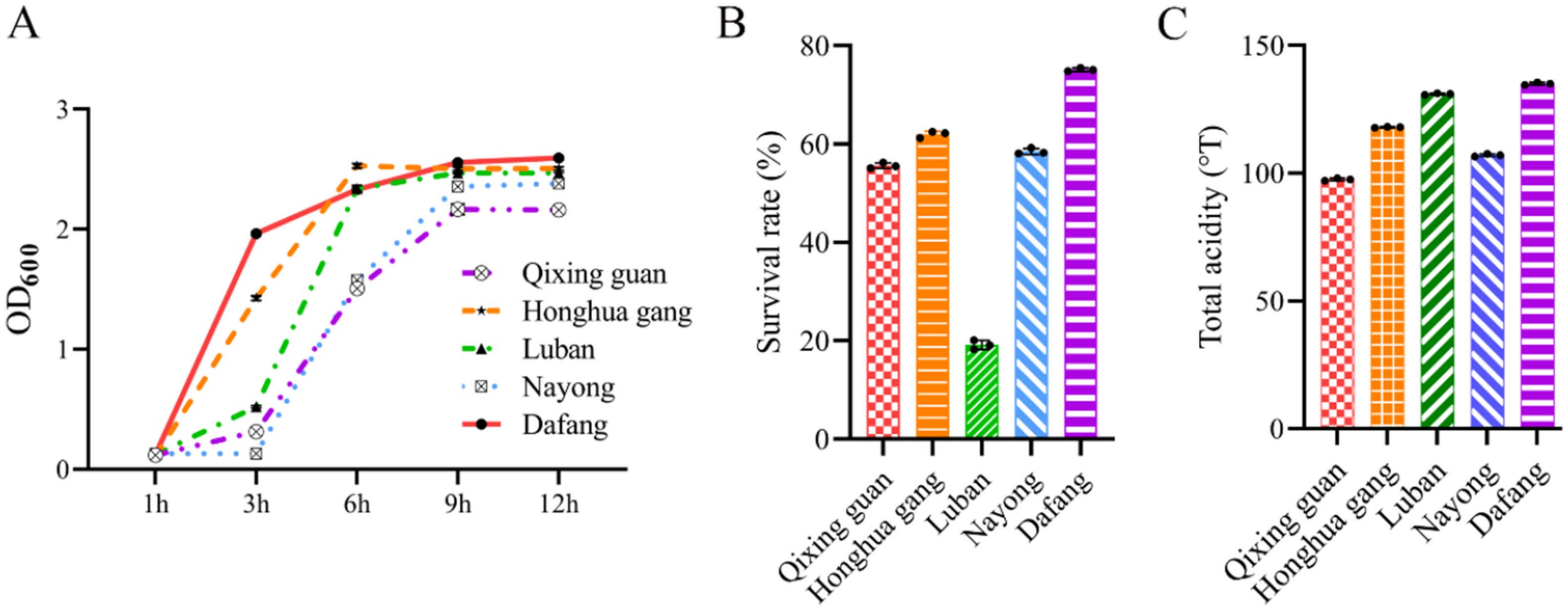
Analysis of the activity and acid production capacity of LAB. (A) LAB growth curve; (B) survival rates of viable bacteria at pH 3 (Lin et al., 2018); (C) acid production ability of LAB strains. 1OD is equivalent to a LAB concentration of 1 × 10^8^ cells/mL. All results are presented as the means ± SD, *n* = 3.

Growth curves showed a fast increase that entered a stable phase after 9 h (at the end of the logarithmic phase) (Figure 2A). Growth was fastest for strains from Dafang and Honghua gang. LAB cultured for 6–9 h were selected to test for acid resistance and production. Except Luban (Figure 2B), the survival rate after acid exposure for all the strains was higher than 50%, with Dafang showing the highest survival rate (> 70%). Dafang strain also showed more acid production than the other strains (Figure 2C).

**Figure 3:**
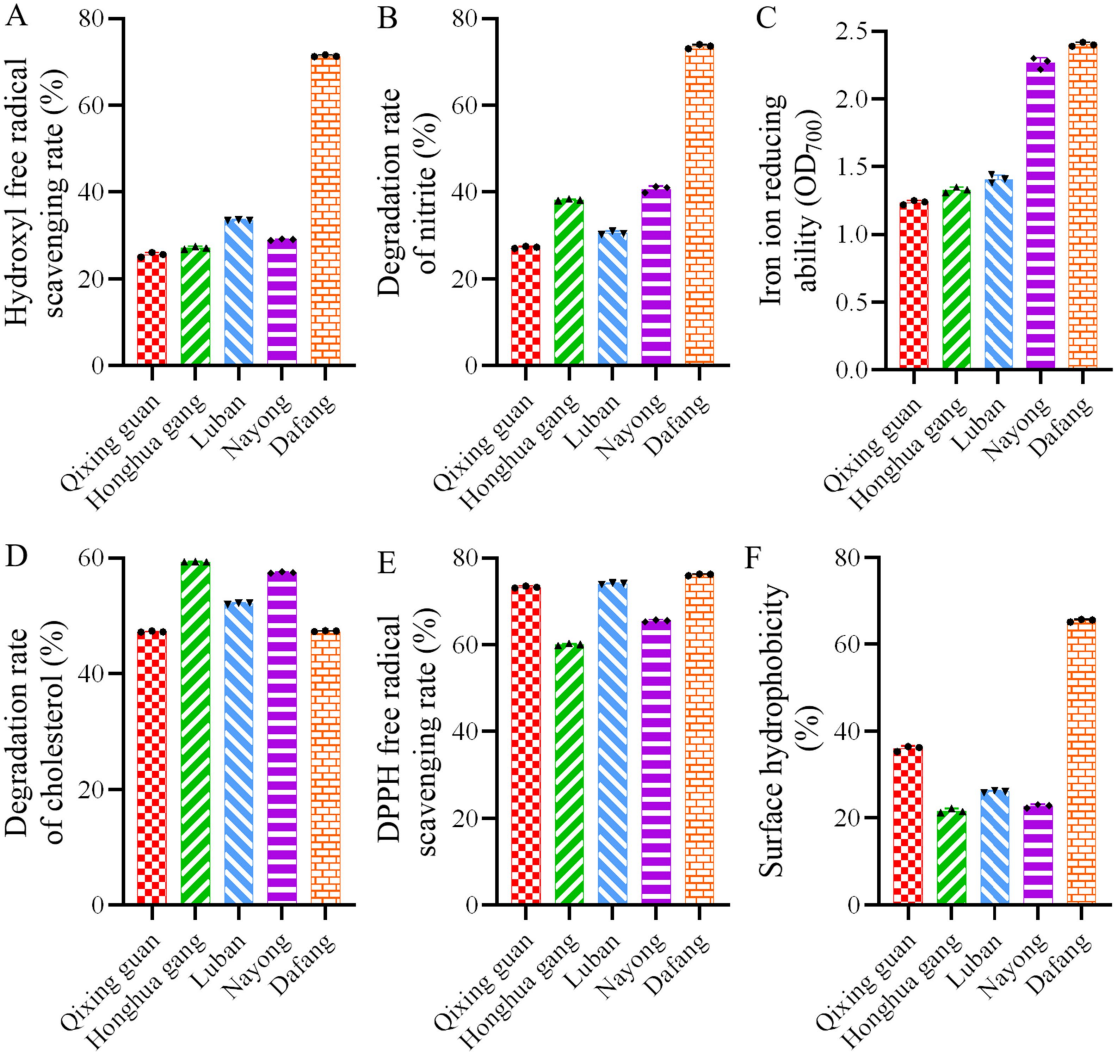
Probiotic analysis of various LAB. (A) Hydroxyl free radical scavenging rate; (B) nitrite degradation rate; (C) Iron ion reducing ability; (D) cholesterol degradation rate; (E) DPPH free radical scavenging rate; (F) surface hydrophobicity. All results are presented as the means ± SD, n = 3.

We then analyzed hydroxyl radicals, DPPH free radicals, cholesterol degradation, nitrite degradation and reduction capabilities (Figure 3). In Dafang LAB, scavenging rate of hydroxyl radicals (Figure 3A) and the degradation rate of nitrite (Figure 3B) were about twice as high than in other regions. LAB isolated from Dafang and Nayong showed more iron reducing power than LAB from other regions (Figure 3C). No significant differences were observed in terms of the DPPH free radical scavenging rate or cholesterol degradation rate (Figures 3D,E), whereas surface hydrophobicity in LAB from Dafang was the highest (Figure 3F). Overall, Dafang LAB has clear advantages, and they were used in further experiments.

### 3.2 Physicochemical functional analysis of Dafang LAB

**Figure 4:**
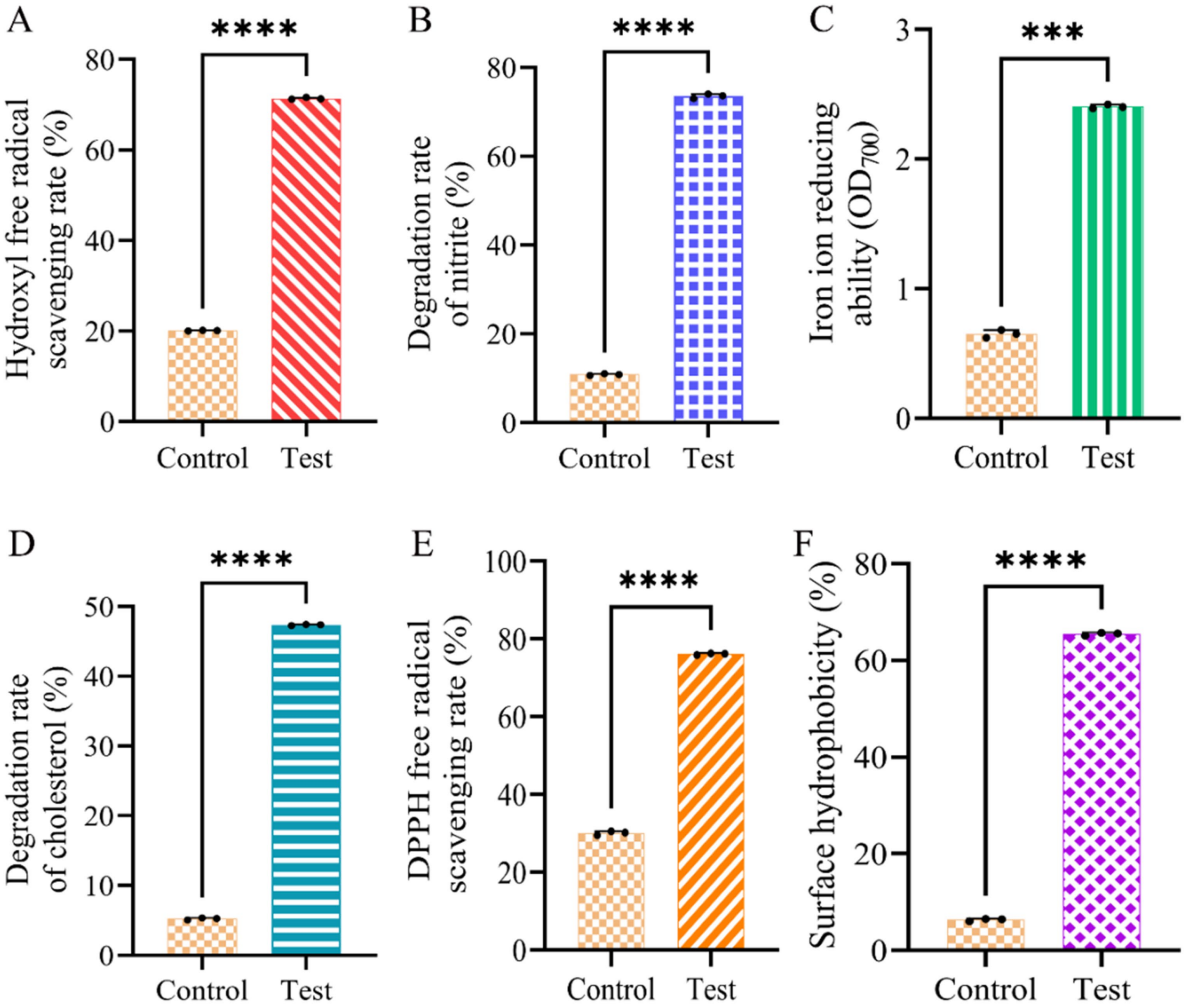
Probiotic analysis of Dafang LAB. (A) Hydroxyl free radical scavenging rate; (B) degradation rate of nitrite; (C) reducing power of iron ion; (D) degradation rate of cholesterol; (E) DPPH free radical scavenging rate; (F) surface hydrophobicity. The control group has no test sample. All data are shown as the means ± S.D. of at least three experiments. The *p*-values were calculated using unpaired Student’s t-tests. No: no significance; *p < 0.05; **p < 0.01; ***p < 0.001.

Based on the above results (Figures 2, 3), Dafang LAB were selected for probiotic analysis after 6 h culture. Compared to the control group, these LAB exhibited higher scavenging rates of hydroxyl radicals (Figure 4A), degradation rate of nitrite (Figure 4B), reducing power for iron (Figure 4C), degradation rate for cholesterol (Figure 4D) and scavenging rate for DPPH free radicals (Figure 4E). Finally, Dafang LAB were more hydrophobic (~10x) than the control group (Figure 4F). Thus, fermentation of Salt-free sauerkraut culture conditions of Dafang LAB may be further optimised.

### 3.3 Optimisation of industrial culture of Dafang LAB

**Figure 5:**
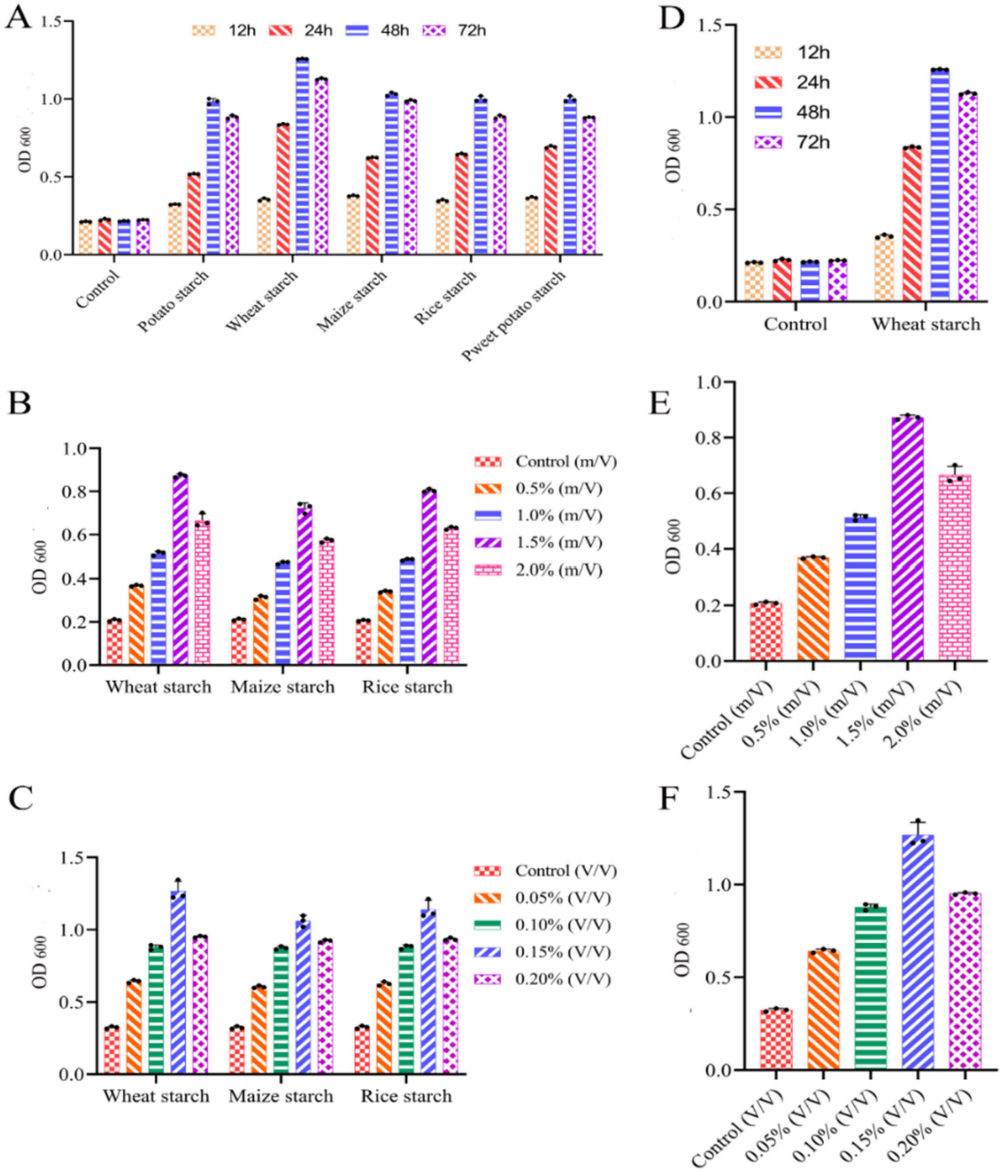
Single factor experimental analysis of LAB. (A) Growth in different starch leaching solutions at different times (1.5% starch leaching solution and 0.1% inoculation); (B) growth with different concentrations of starch extracts in a 48 h culture; (C) growth with different inoculum in a 48 h culture and 1.5% starch extract; (D) Growth vs. time in culture medium containing 1.5% wheat extract; (E) growth in different contents of wheat extract; (F) growth vs. volume of bacteria. Control measurements used culture medium without inoculation. LAB growth was measured at 600 nm, where 1 OD is equivalent to 1 × 10^8^ cells/mL. All results are presented as means ± SE n = 3.

Culture conditions of Dafang LAB were optimized using media supplemented with different starch extracts and under various culture durations. The highest LAB content was achieved after 48 h, regardless of the type of starch extract (Figure 5A). In wheat starch extract, LAB content at 48 h (Figure 5D). LAB content was highest when surface-to-water ratio was 1.5% (Figures 5B,E). Similarly, LAB cultured in wheat starch extract performed better in rice or corn starch (Figures 5C,F) and was optimal with an inoculum of 0.15%. Then, we investigated the relationships between various factors using three experimental methods: Plackett-Burman (PB), steepest climbing and central composite design (CCD) experiments.

### 3.4 Results and analysis of central composite design

Firstly, the Plackett-Burman experimental design consisted of 12 trials with two levels for each variable (Supplementary Tables S1, S3). To determine the optimal response, a first-order model for LAB production was fitted using the Plackett-Burman experimental design, following Equation 1:


(1) $$\begin{array}{l} Y\;\left({OD}_{600}\;\mathrm{of}\;\mathrm{LAB}\right)=1.82+0.3183^{*}\;A-0.1817^{*}\;B–\\ 0.2117^{*}\;C\;\left(R2=0.9139\right)\text{.} \end{array}$$

The effect of each variable on LAB yield was determined from the coefficients of Equation 1 and the statistical analysis (Supplementary Table S4). Variables with a confidence greater than 95% (*p* < 0.05) were considered significant and were selected for further study. The model showed a linear regression coefficient R^2^ of 0.9139, with *p* value of 0.0001 (*p* < 0.001), suggesting the design was appropriate. The *p* values for culture time (A) (*p* = 0.0001), starch/water ratio (B) (*p* = 0.0042) and inoculum (C) (*p* = 0.0017) were all less than 0.05, corresponding to a 95% confidence level. The lack-of-fit value of the model was not significant (*p* = 0.5301), suggesting a good fit of the model. Thus, variables in A-C were used in subsequent experiments. It is currently accepted that cereals provide a favourable substrate for LAB growth (Dulf et al., 2022; Mendes et al., 2021).

Secondly, Equation 1 show that A coefficient is positive, whereas B and C are negative, suggesting that LAB production should increase by increasing culture time and decreasing starch/water ratio and inoculum (Supplementary Table S2). To determine the best direction change for these three factors while keeping other factors constant in the basal culture medium, the path of steepest ascent was used, where the highest LAB yield was achieved when culture time, starch/water ratio and inoculum were 48 h, 1.5 and 0.015%, respectively (Supplementary Table S5).

Finally, the interaction between the three factors described above was analysed using central composite design and response surface methodology. The optimal level for these variables was determined using the values obtained from the steepest ascent path as centre points (Table 1, Supplementary Tables S2, S5), while keeping the other variables fixed at a low level (Table 3).

**Table 3:** Design and results of central composite approach.

Run	Culture time (h)	Starch/water ratio (m/V)	(V/V)	LAB yield (OD_600_)
1	0.000	0.000	0.000	2.86
2	0.000	1.682	0.000	1.96
3	1.000	−1.000	1.000	1.71
4	0.000	−1.682	0.000	1.45
5	−1.000	1.000	−1.000	0.88
6	0.000	0.000	0.000	2.79
7	−1.000	−1.000	1.000	1.63
8	1.000	−1.000	−1.000	1.33
9	1.000	1.000	1.000	1.99
10	1.682	0.000	0.000	1.56
11	0.000	0.000	0.000	2.84
12	1.000	1.000	−1.000	1.92
13	−1.000	1.000	1.000	1.89
14	−1.000	−1.000	−1.000	0.89
15	0.000	0.000	0.000	2.81
16	0.000	0.000	0.000	2.78
17	−1.682	0.000	0.000	1.01
18	0.000	0.000	0.000	2.83
19	0.000	0.000	−1.682	0.46
20	0.000	0.000	1.682	1.52

The results were subjected to analysis of variance (ANOVA) on Design Expert 13, and the resulting regression model is represented by the following equation:


(2) $$\begin{array}{l} Y\;\left(\begin{array}{l} {OD}_{600}\;\mathrm{of}\;\\ \mathrm{LAB} \end{array}\right)=2.86329+0.200265^{*}\;A+0.147011^{*}\;B+\\ 0.296453^{*}\;C+0.06375^{*}\;\mathrm{AB}-\\ 0.19125^{*}\;\mathrm{AC}-0.01875^{*}\;BC-\\ 0.506218^{*}\;A^{2}-0.357726^{*}\;B^{2}-\\ 0.601678^{*}\;C^{2}-\\ 0.06875^{*}\;ABC\;\left(R^{2}=0.9730\right)\text{.} \end{array}$$

The relationships between LAB production (Y) and culture time (A), starch/water ratio (B) and inoculum concentration (C) are described in Table 1. The ANOVA of the quadratic regression model (Equation 2) demonstrated that this is highly significant model. Indeed, Fisher’s F test yielded a very low probability value (*F* value = 34.42) (Table 4), whereas the p value (<0.0001) was less than 0.01% with 99% confidence and the lack-of-fit was not significant (F value of 3.14; *p* = 0.1205).

**Table 4:** Analysis of variance (ANOVA) for regression of central composite design.

Source	Sum of squares	df	Mean square	F value	*p* value	
Model	11.50	10	1.15	32.42	< 0.0001	****
A-A (Culture time)	0.5477	1	0.5477	15.44	0.0035	**
B-B (Starch/water ratio)	0.2952	1	0.2952	8.32	0.0180	*
C-C (Inoculum)	1.20	1	1.20	33.83	0.0003	***
AB	0.0325	1	0.0325	0.9165	0.3634	
AC	0.2926	1	0.2926	8.25	0.0184	*
BC	0.0028	1	0.0028	0.0793	0.7846	
A^2^	3.69	1	3.69	104.10	< 0.0001	****
B^2^	1.84	1	1.84	51.98	< 0.0001	****
C^2^	5.22	1	5.22	147.06	< 0.0001	****
ABC	0.0378	1	0.0378	1.07	0.3288	
Residual	0.3193	9	0.0355			
Lack of fit	0.2284	4	0.0571	3.14	0.1205	
Pure error	0.0909	5	0.0182			
Cor Total	11.82	19				

*p < 0.05, **p < 0.01, ***p < 0.001, ****p < 0.0001.

Culture time (A, *p* = 0.0035), starch/water ratio (B, *p* = 0.0180) and inoculum (C, *p* = 0.0003) had significant effects on LAB yield, and interaction between culture time (A) and inoculum (C) also was significant (*p* = 0.0184). The quadratic terms (A^2^, B^2^, C^2^) were also significant (*p* > 0.05), and the lack-of-fit value of the model was not significant (*p* = 0.1205). The “predicted R^2^” of this model was 0.9730, in reasonable agreement with the adjusted R^2^ of 0.9430. Adequate precision, measured by the signal-to-noise ratio, is desirable when the value is 4, whereas our model achieved a ratio of 15.75. The coefficient of variation of the model was 2.86, which indicated a high reliability.

To determine the optimum LAB production for the selected variables, response surface plots were analysed to solve the regression equation obtained after ANOVA. This equation provides an estimate of LAB production based on culture time, starch/water ratio, and inoculum. The experimental design developed in this study was more accurate at optimising the components of closed media for LAB production. Based on the above analysis, the nonsignificant items in the t-test were deleted, and Equation 2 was further optimised as follows:


(3) $$\begin{array}{l} Y\;\left(\begin{array}{l} {OD}_{600}\;\mathrm{of}\;\\ \mathrm{LAB} \end{array}\right)=2.86329+0.200265^{*}\;A+0.147011^{*}\;B+\\ 0.296453^{*}\;C-0.19125^{*}\;\mathrm{AC}-\\ 0.506218^{*}\;A^{2}–0.357726^{*}\;B^{2}–\\ 0.601678^{*}\;C^{2}\;\left(R^{2}=0.9730\right)\text{.} \end{array}$$

**Figure 6:**
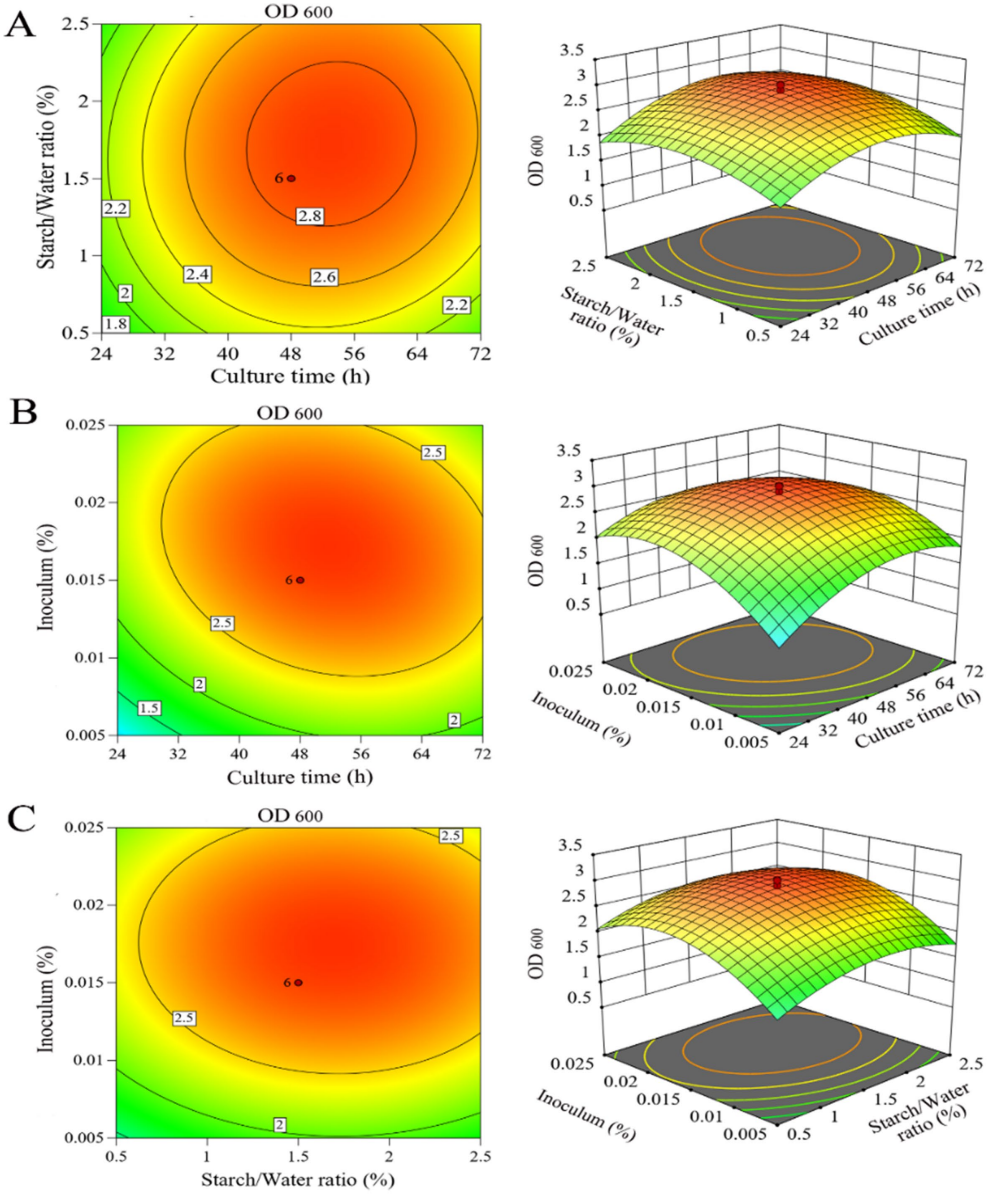
Three-dimensional response surface analysis and contour plots for LAB. (A) Effect of culture time and starch/water ratio when inoculum is 0.015%; (B) effect of culture time and inoculum when starch/water ratio level is 1.5%; (C) effect of starch/water ratio and inoculum when culture time is 48 h.

The interactions of the three components and their optimum level for LAB production were analysed using response surface methodology (Figure 6). To generate the three-dimensional graphs, two variables were combined while keeping the other variable at the optimum level determined by the path of steepest ascent for LAB production. The response surface was convex, which indicated well-defined optimum conditions and a maximum value for each variable. Based on these results, the predicted maximum production of LAB was 2.6 when culture time, starch/water ratio and inoculum were set at 66 h, 1.2 and 0.02%, respectively, while the remaining variables were kept at standard levels.

**Figure 7:**
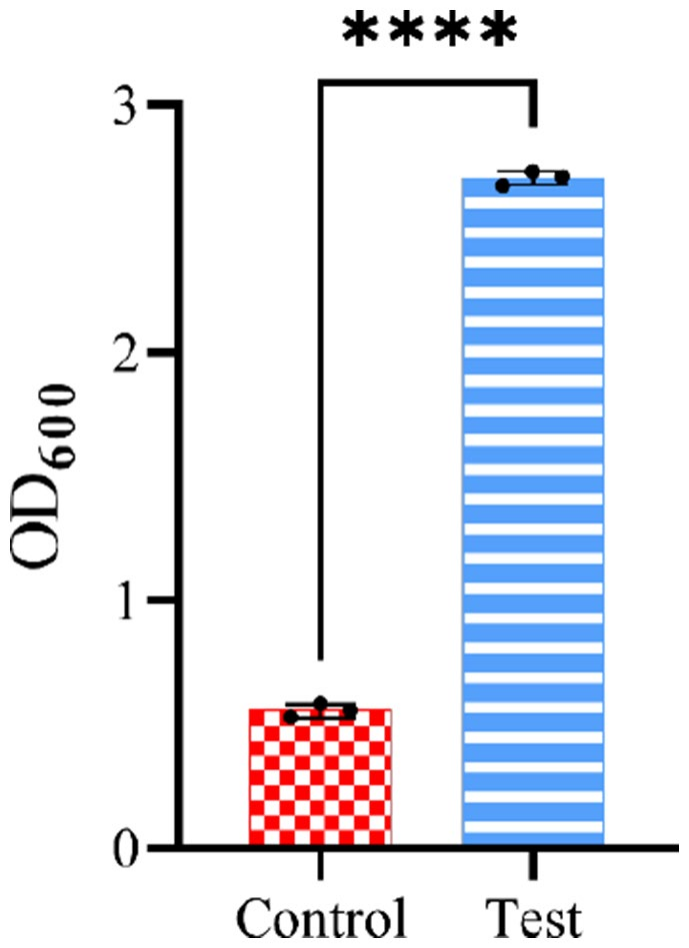
Experimental verification of CCD optimisation results. The control group is the non-optimised culture medium, while the test group used the medium conditions optimised by CCD.

To evaluate the accuracy of the model in (Equations 2, 3) predicting optimum response values, LAB were cultivated in an optimised medium, where the yield obtained was 2.705, close to the predicted value 2.618. Notably, the use of the optimised culture medium resulted in an approximately 5-fold increase in LAB production compared to the basal culture medium (Figure 7).

### 3.5 Small-scale fermentation of salt-free sauerkraut and physicochemical characterization

**Figure 8:**
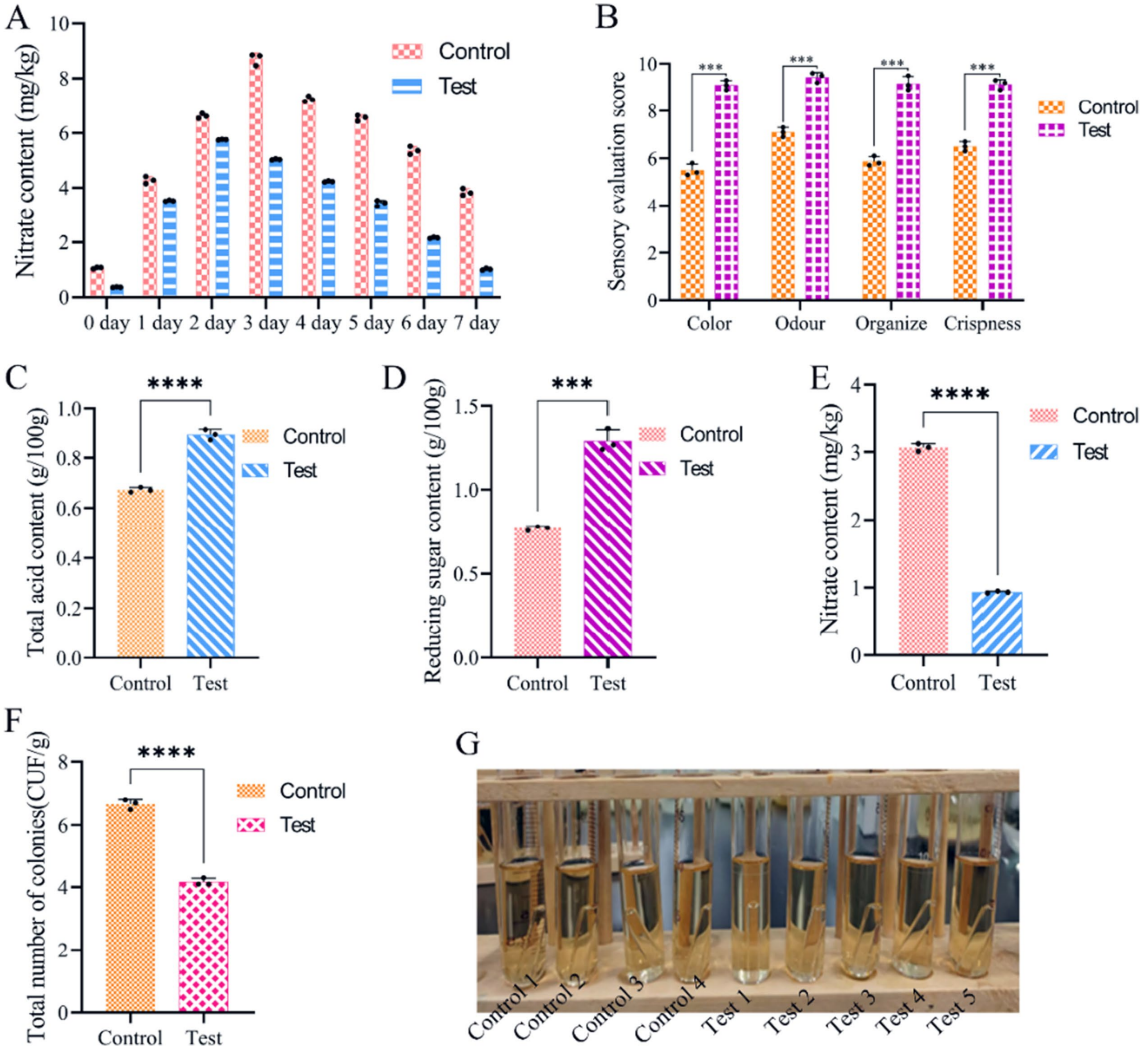
Analysis of salt-free sauerkraut fermentation. (A) Change of nitrite content; (B) sensory evaluation; (C) total acid content; (D) reducing sugar content; (E) nitrite content; (F) total microbial colonies; (G) detection and analysis of *E. coli* bacteria according to national standards. The control was naturally fermented sauerkraut without salt.

To evaluate the impact of the LAB obtained via CCD optimisation on the fermentation of salt-free sauerkraut, we followed the flow outlined in Figure 1. We found that during fermentation, nitrite content gradually decreased from 48 h to 7 days (Figure 8A). The sensory properties of the sauerkraut were evaluated after simulated pasteurisation. Sauerkraut without HCl had a sensory score of 36.82/40, 32% higher than the control with HCl (Figure 8B). Also, total acid and reducing sugar content increased by 32.54% (Figure 8C) and 67.27% (Figure 8D), respectively, whereas nitrite decreased by 69.58% (Figure 8E). The total number of bacterial colonies decreased by 37.5% (Figure 8F) and no coliform bacteria was detected in the final product, with no bubbles produced in neither of the nine test tubes (Figure 8G). The probable number of flora, as determined by the coliform group (MPN) index table, was ≤30 MPN/100 g, which complies with the national limit for coliform bacteria in pickled vegetables in bags. Collectively, these findings indicate that pasteurised, bagged salt-free fermented sauerkraut is unlikely to contain coliform bacteria.

## 4 Discussion

Recently, salt-free sauerkraut has gained popularity due to its low salt, sugar and having acidity. Herein we successfully isolated a strain of Dafang LAB from salt-free sauerkraut used to ferment sour soup. Through optimization of culture conditions, we show that Dafang LAB fulfills current market requirements for sauerkraut products.

LAB play a crucial role in the fermentation of sauerkraut (Wang J. et al., 2019; Liu et al., 2016; Cani et al., 2022; Van Zyl et al., 2020; Tlais et al., 2022), with Lactobacillus species identified as dominant, particularly in the Northeast region (Wang W. et al., 2019; Sun et al., 2022; Wang et al., 2017; Cong et al., 2016), as well as in other locations (Shu et al., 2019; Cheng et al., 2022; Feng et al., 2021; Yuan et al., 2019; Li et al., 2012, 2014; Zhao et al., 2020; Zhang et al., 2017; Cao and Zhang, 2017; Huang et al., 2023).These lactobacilli possess good acid resistance, acid production, nitrite degradation ability, salt resistance, bile salt resistance, bacteriostatic properties and temperature sensitivity (Wang W. et al., 2019; Sun et al., 2022; Wang et al., 2017; Cong et al., 2016; Shu et al., 2019; Cheng et al., 2022; Feng et al., 2021; Yuan et al., 2019; Li et al., 2012, 2014; Zhao et al., 2020; Cao and Zhang, 2017; Huang et al., 2023). However, they have not been identified in salt-free sauerkraut in Guizhou.

The isolated Dafang LAB we isolated is similar to the one isolated previously (Wang et al., 2022). The probiotic properties of LAB have been shown before (Lee et al., 2022; Xia et al., 2022), especially the ability to reduce cholesterol and nitrite levels and antioxidant properties (Yang et al., 2022). LAB also regulate microbial flora, alleviates lactose intolerance and enhances immune function.

The selected Dafang LAB has more than three times higher antioxidant and reducing capacity than the control group, and higher antioxidant capacity than LAB in Northeastern sauerkraut (Zou et al., 2023). Also, Dafang LAB exhibit cholesterol and nitrite degradation more than six times greater than the control group. This is consistent with observations in the Samburu tribe in Africa (Moiseenko et al., 2021), where a high consumption of dairy products fermented by wild Lactobacillus was associated with a reduction in cholesterol levels (Yang et al., 2021). Safety for consumption and cholesterol absorption was confirmed previously (Wan et al., 2021).

A key factor for bacteria to colonise hosts is surface adherence (Feng et al., 2019), where hydrophobicity overcomes electrostatic repulsion. Adhesion requires formation of chemical bonds between ligands and receptors on mucosal epithelial cells, and understanding these interactions at the molecular level is essential to study Lactobacillus probiotic colonisation of the intestinal tract.

Salt-free sauerkraut fermentation also requires optimising culture conditions of LAB. To address the problems that affect flavour and taste in salt-free fermented sauerkraut, we cultivated LAB in various starch leaching solutions (Hatti-Kaul et al., 2018; Zuo et al., 2022). Wheat starch was selected, and CCD technology was used to fully optimise culture conditions which were consistent with previous studies (Zhang et al., 2017), solving the problems encountered in the fermentation process of salt-free sauerkraut, such as turbidity of the bacterial liquid, excessive suspended matter, and unstable taste of salt-free sauerkraut (Hatti-Kaul et al., 2018; Zuo et al., 2022), and production of LAB herein was more than 2–5 times greater. Finally, Dafang LAB used in a pilot experiment involving salt-free sauerkraut fermentation.

It is generally accepted that LAB are crucial for the vegetable fermentation transition from traditional natural methods to modern purebred fermentation. The latter should result in rapid, stable and high-quality fermentation of salt-free sauerkraut (Xu et al., 2022). Purebred microorganisms (Yujian et al. 2013) help to produce low-salt, low-nitrite, fresh, crunchy salt-free sauerkraut products (Wang et al., 2020), and our small-scale industrial test demonstrate lower nitrite content, lower total acid content and higher reducing sugar content than the control group.

Sensory evaluation of the sauerkraut product without salt also improved, suggesting a role for LAB in the fermentation of salt-free sauerkraut. This finding is consistent with previous reports (Ye et al., 2018) that found that dominant bacteria involved in salt-free sauerkraut fermentation contributed to its overall mouthfeel, either by single (He J. et al., 2021) or mixed (Jing et al., 2016) LAB. Salt-free sauerkraut meets the national standard and fulfill market requirements, as evidenced by the total colony count and absence of coliform bacteria.

The importance of food taste cannot be overestimated. In China, sauerkraut is a widely enjoyed fermented vegetable known for its crisp texture and sour taste (Yun et al., 2021; Hu et al., 2022). It is claimed that it stimulates appetite, aids digestion and facilitates the absorption of essential nutrients. The successful implementation of this project will contribute to the promotion of salt-free sauerkraut within the green food industry while emphasizing its low salt, low sugar, and increased acidity characteristics.

We highlight the crucial role of LAB in the fermentation of salt-free sauerkraut, offering new possibilities for the cultivation and innovation of strains used in salt-free sauerkraut technology, providing a basis for its introduction to the market.

## Data Availability

The original contributions presented in the study are included in the article/Supplementary material, further inquiries can be directed to the corresponding author.

## Footnotes

^1^http://down.foodmate.net/standard/sort/3/50419.html

^2^http://down.foodmate.net/standard/sort/3/16237.html

^3^http://down.foodmate.net/standard/sort/3/11619.html

^4^http://down.foodmate.net/standard/sort/3/50368.html

^5^http://down.Foodmate.net/standard/sort/3/50367.html

^6^http://down.foodmate.net/standard/sort/3/145883.html

^7^https://www.Bzwz.com/s_62/s_10193.html

^8^http://down.foodmate.net/standard/sort/15/41770.html
