# Supplementary material for: Optimisation of Lactobacillus fermentation conditions and its application in the fermentation of salt-free sauerkraut
Source: Front Microbiol. 2024 Oct 21;15:1482163. doi: 10.3389/fmicb.2024.1482163 (PMC11532087; doi:10.3389/fmicb.2024.1482163)
Supplement: Supplementary file 1 [file Data_Sheet_1.PDF]

# Supplementary Materials for
# Optimisation of Lactobacillus fermentation conditions and its application in the fermentation of salt-free sauerkraut

This PDF file includes: Figure S1

Plackett-Burman experimental design

Path of steepest ascent method

Experiment Results and Analysis of Plackett-Burman (PB), Table S1- Table S4.

Experiment Results and Analysis of Path of steepest ascent，Table S5；

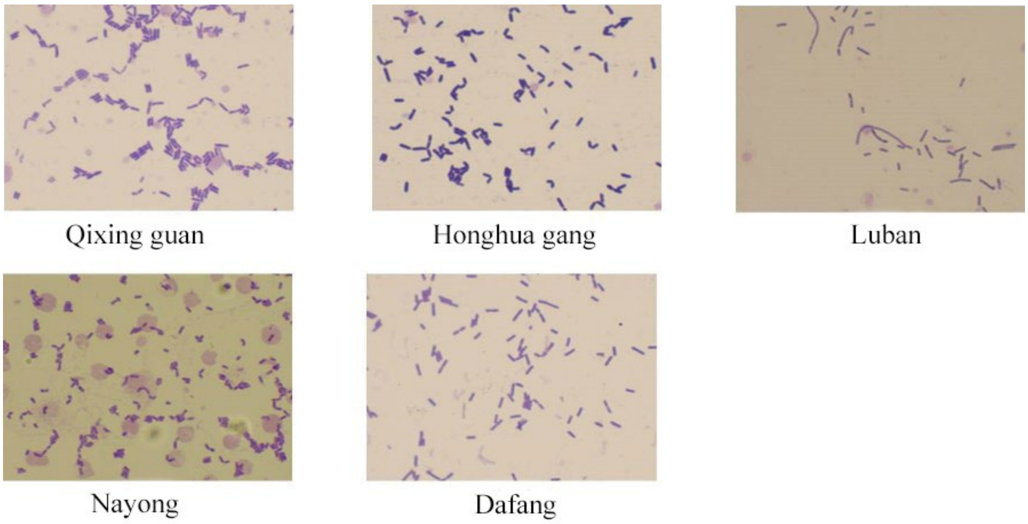


Figure S1 Gram staining and morphology of LAB isolated from the five locations indicated.

***Plackett-Burman experimental design***

The effect of culture time, starch/water ratio and inoculum on the yield of LAB cultivated in 100-mL Erlenmeyer flasks, we used single-factor experiments performed in triplicate, using the average LAB yield as the response. Fermentation time for each experiment was two days, and experiments were performed as described previously. A Plackett-Burman experimental design was used for initial screening of the medium and culture conditions for all three components. Each variable was assigned two levels (Table 1): low level (code -1) and high level (code 1). To examine the experimental design, two dummy variables (X4 and X11) were used and fitted into a first-order model.

$$Y = \alpha_0 + \sum \alpha_i X_i \ (i = 1,2, \cdots k) \quad (1)$$

where $Y$ is the estimated target function, $\alpha_0$ is the model intercept, $\alpha_i$ is the regression coefficient, and $X_i$ is the coded independent factor.

**Table S1** Plackett-Burman design to screen the variables

| Variable | Factor | Low level (-1) | High level (1) |
|---|---|---|---|
| $X_1$ | Culture time (h) | 24 | 72 |
| $X_2$ | Starch/Water ratio (%) | 1 | 2 |
| $X_3$ | Inoculum (%) | 0.1 | 0.2 |
| $X_4$ - $X_{11}$ | Dummy variable | -1 | 1 |

- *Path of steepest ascent method*

Using the findings from the Plackett-Burman experimental design, examination of the optimal level for each chosen factor was performed using the steep ascent approach. Using the direction of the Plackett-Burman experimental value as the upwards inclination, the design of the steep ascent method was established. The adjustment in step size was validated based on the data from the Plackett-Burman experimental design. Whenever the variable effect was positive, higher values were chosen, and viceversa (Table 2).

**Table S2** Steepest ascent experimental design

| Experiment number | Culture time (h) | Starch/Water ratio (%) | Inoculum (%) |
|---|---|---|---|
| 1 | 24 | 2.5 | 0.025 |
| 2 | 36 | 2.0 | 0.020 |
| 3 | 48 | 1.5 | 0.015 |
| 4 | 60 | 1.0 | 0.010 |
| 5 | 72 | 0.5 | 0.005 |

**Table S3** Plackett-Burman design and results.

| Run | Culture time (h) | Starch/water ratio (m/V) | Inoculum (V/V) | LAB yield ($OD_{600}$) |
|---|---|---|---|---|
| 1 | 1 | -1 | 1 | 2.23 |
| 2 | -1 | 1 | 1 | 1.09 |
| 3 | 1 | 1 | 1 | 1.97 |

| | | | | |
|---|---|---|---|---|
| 4 | -1 | 1 | -1 | 1.42 |
| 5 | 1 | -1 | -1 | 2.55 |
| 6 | -1 | -1 | 1 | 1.49 |
| 7 | 1 | 1 | -1 | 2.18 |
| 8 | 1 | -1 | 1 | 1.79 |
| 9 | -1 | 1 | 1 | 1.06 |
| 10 | 1 | 1 | -1 | 2.09 |
| 11 | -1 | -1 | -1 | 1.92 |
| 12 | -1 | -1 | -1 | 2.01 |

Note: 1OD is equivalent to a LAB concentration of $1*10^8$ cells/mL

**Table S4 Statistical analysis of Plackett-Burman results.**

| Source | Sum of Squares | df | Mean Square | F value | p value | |
|---|---|---|---|---|---|---|
| Model | 2.15 | 3 | 0.7166 | 28.30 | 0.0001 | *** |
| A-A (Culture time) | 1.22 | 1 | 1.22 | 48.03 | 0.0001 | *** |
| B-B (Starch/Water ratio) | 0.3960 | 1 | 0.3960 | 15.64 | 0.0042 | ** |
| C-C (Inoculum) | 0.5376 | 1 | 0.5376 | 21.23 | 0.0017 | *** |
| Residual | 0.2026 | 8 | 0.0253 | | | |
| Lack of Fit | 0.0972 | 4 | 0.0243 | 0.9228 | 0.5301 | |
| Pure Error | 0.1053 | 4 | 0.0263 | | | |
| Cor Total | 2.35 | 11 | | | | |

* = p<0.05, ** = p<0.01, *** = p<0.001, **** = p<0.0001.

**Table S5** Design and results of path of steepest ascent method.

| | Culture time (h) | Starch/Water ratio (%) | Inoculum (%) | LAB Yield ($OD_{600}$) |
|---|---|---|---|---|
| 1 | 24 | 2.5 | 0.025 | 1.88 |
| 2 | 36 | 2.0 | 0.020 | 2.23 |
| 3 | 48 | 1.5 | 0.015 | 2.84 |
| 4 | 60 | 1.0 | 0.010 | 2.16 |
| 5 | 72 | 0.5 | 0.005 | 1.63 |
